# Smoking prevalence, readiness to quit and smoking cessation in HIV+ patients in Germany and Austria

**DOI:** 10.7448/IAS.17.4.19729

**Published:** 2014-11-02

**Authors:** Olaf Degen, Peter Arbter, Peter Hartmann, Christoph Mayr, Thomas Buhk, Horst Schalk, Helmut Brath, Thomas Ernst Dorner

**Affiliations:** 1 Universitätsklinikum Hamburg-Eppendorf, Ambulanzzentrum, Hamburg, Germany; 2 Praxis Arbter, Krefeld, Germany; 3 Praxisplus, Münster, Germany; 4 Ärzteforum Seestrasse, Berlin, Germany; 5 Infektionsmedizinisches Centrum Hamburg, Hamburg, Germany; 6 Schalk:Pichler Gruppenpraxis, Wien, Austria; 7 Diabetesambulanz, Gesundheitszentrum Süd, Wiener Gebietskrankenkasse, Wien, Austria; 8 Centre for Public Health, Medical University Vienna, Wien, Austria

## Abstract

**Introduction:**

Due to the interaction between smoking and the virus and the antiretroviral therapy, the excess health hazard due to smoking is higher in HIV+ patients than in the general population. International studies suggest a higher prevalence of smoking in HIV+ subjects compared to the general population. It was the aim of the study to assess prevalence of smoking, to analyze determinants of smoking, and to evaluate readiness to quit in HIV+ patients in Germany and Austria.

**Material and Methods:**

Consecutive patients with positive tested HIV status, smokers and non-smokers, who are treated in seven different HIV care centres in Austria and Germany were included. Nicotine dependence was assessed with the Fagerström Test for Nicotine Dependency (FTND), and stages of change by a standardized readiness to quit questionnaire. Self-reported smoking status was objectified by measuring exhaled carbon monoxide levels. Smokers who wanted to quit were offered a structured smoking cessation programme, and those who did not want to quit received a 1-minute consultation. After six months, the smoking status of all included subjects was reassessed.

**Results:**

A total of 447 patients were included; the response rate was 92%. Prevalence of smoking was 49.4%. According to a multivariate logistic regression analysis, lower age, male sex, lower educational level, and smoking of the partner were significantly associated with the smoking status. According to the FTND, 25.3% showed a low (0–2 points), 27.6 a moderate (3–4 points) and 47.1% a high (5–10 points) dependency. Regarding stages of change, 15.4% of the smokers were in the stadium precontemplation, 48.4 in contemplation, 15.4 in preparation and 10.0 in the stadium action. 11.0% were not assignable in any stadium. Higher education level and lower grade of dependency were significantly associated with the wish to quit smoking. Six months after the baseline examination, smoking cessation visits (at least one session) was performed in 28.5% of the smokers. 13% of the smokers have quit smoking, 23% have reduced smoking and 63% did not change smoking habits positively 6 months after the first visit.

**Conclusions:**

Prevalence rates for smoking in HIV+ subjects are higher than in the general population. Readiness to quit is, however, high, and 13% of smokers who have quit smoking after six months is a remarkable short-term success. This observation underlines the importance and feasibility of addressing smoking habits in HIV care.

